# Smartphone-Based Electrochemical Systems for Glucose Monitoring in Biofluids: A Review

**DOI:** 10.3390/s22155670

**Published:** 2022-07-28

**Authors:** Jie Xu, Zupeng Yan, Qingjun Liu

**Affiliations:** Biosensor National Special Laboratory, Key Laboratory for Biomedical Engineering of Education Ministry, Department of Biomedical Engineering, Zhejiang University, Hangzhou 310027, China; xujiezju@zju.edu.cn (J.X.); 21734016@zju.edu.cn (Z.Y.)

**Keywords:** glucose sensor, electrochemical detection, smartphone, wearable monitoring, implantable monitoring, self-power

## Abstract

As a vital biomarker, glucose plays an important role in multiple physiological and pathological processes. Thus, glucose detection has become an important direction in the electrochemical analysis field. In order to realize more convenient, real-time, comfortable and accurate monitoring, smartphone-based portable, wearable and implantable electrochemical glucose monitoring is progressing rapidly. In this review, we firstly introduce technologies integrated in smartphones and the advantages of these technologies in electrochemical glucose detection. Subsequently, this overview illustrates the advances of smartphone-based portable, wearable and implantable electrochemical glucose monitoring systems in diverse biofluids over the last ten years (2012–2022). Specifically, some interesting and innovative technologies are highlighted. In the last section, after discussing the challenges in this field, we offer some future directions, such as application of advanced nanomaterials, novel power sources, simultaneous detection of multiple markers and a closed-loop system.

## 1. Introduction

As an important source of carbon and energy, glucose is a biomarker of many diseases, and glucose monitoring in different biofluids is of great significance in clinical practice. Induced by decreased insulin secretion or insulin resistance, diabetes is a group of metabolic disorders characterized by increased blood glucose level. The increase of blood glucose level causes chronic damage to vessels and nerves, resulting in the injury of multiple organs and even acute coma and death. Hence, blood glucose level is not only an important marker for the diagnosis of diabetes, but also a major indicator for diabetes management [1,2,3]. In addition, glucose is a biomarker of peritoneal carcinomatosis and peritonitis. The value of glucose in these diseases has been investigated since the 1970s and used in clinical practice. According to the hypothesis in the reports, peritoneal glucose level fluctuates with blood glucose concentration in healthy conditions. Nevertheless, for peritoneal carcinomatosis and peritonitis patients, the tumor cells or microorganisms in their peritoneal cavities uptake glucose in ascites and result in a lower glucose concentration in ascites [4,5,6].

Electrochemical detection is a technology that analyses and measures substances according to their electrochemical characterizations. It can evaluate the concentration of analytes by applying an input of electrical energy to the electrochemical electrode and recording its responses of current, potential or resistance [7]. The electrochemical detection method mainly includes chronoamperometry, cyclic voltammetry, square wave voltammetry and differential pulse voltammetry. For example, chronoamperometry applies a step voltage and then a constant voltage to an electrode and detects its current response. In recent years, electrochemical detection systems have been shown to be powerful tools that are efficiently applied in various fields because of their great sensitivity, low cost, portability and ease of operation [8]. When sensing glucose using the electrochemical method, glucose is oxidized via glucose oxidase, glucose dehydrogenase or non-enzymatic catalysts, electrons are transferred on the electrode surface, and an electrical signal is generated [9,10,11,12]. These three types of catalysts for glucose sensing have their advantages and drawbacks. The main advantage of glucose oxidase is high selectivity. However, it is not stable and is susceptible to temperature, humidity and pH. Glucose dehydrogenase has higher activity than glucose oxidase [13]. However, its selectivity is not ideal and glucose sensors based on it could be interfered with by maltose, galactose or xylose [14,15]. Non-enzymatic glucose detection is based on metal redox centers, mainly including noble metals, transition metals and metallic alloy. The noble metal and alloy-based sensors can operate well in neutral pH conditions, which is the physiological pH condition of most biofluids. However, they show inferior sensitivity, anti-interference and stability compared to transition metal counterparts. While the transition metal-based sensors show superior sensitivity and anti-interference compared to the other sensor types, they perform poorly in neutral pH [16].

Since Clark and Lyons developed the first electrochemical electrode in 1962, electrochemical glucose detection has developed rapidly; indeed, electrochemical glucose detection forms are changing from large-scale to point-of-care with the rapid development of mechanical design, wireless technology, low-power electronics, manufacturing processes, flexible electronics technology and functional nanomaterials [17,18,19,20]. A point-of-care blood glucose monitoring device with enzyme-based glucometers and disposable strips was developed and then used widely for self-monitoring of blood glucose [21,22,23]. For example, the Abbott company produced a portable blood glucose testing device, with the advantages of small size and convenient use. The testing only needs a drop of blood sample and could be completed in seconds. This portable device can help patients evaluate blood glucose level and adjust the treatment strategy in time [24].

In the development of point-of-care detection, smartphones have played an important role. Smartphones are now becoming one of the most widely used mobile devices. The ubiquitous availability guarantees a convenient and cost-effective acquisition of smartphones [25]. With multi-network access, data analysis capability, screen display and an open-source operation system, smartphones enable a point-of-care test outside of laboratories with an efficient simplified electronic design, minimizing the size of monitoring systems and reducing their overall cost. Therefore, the wide usage and excellent functions of smartphones in the field of electrochemical glucose detection are inspiring the surge of peripheral apparatus linked to smartphones for real-time glucose monitoring [18].

**Figure 1:**
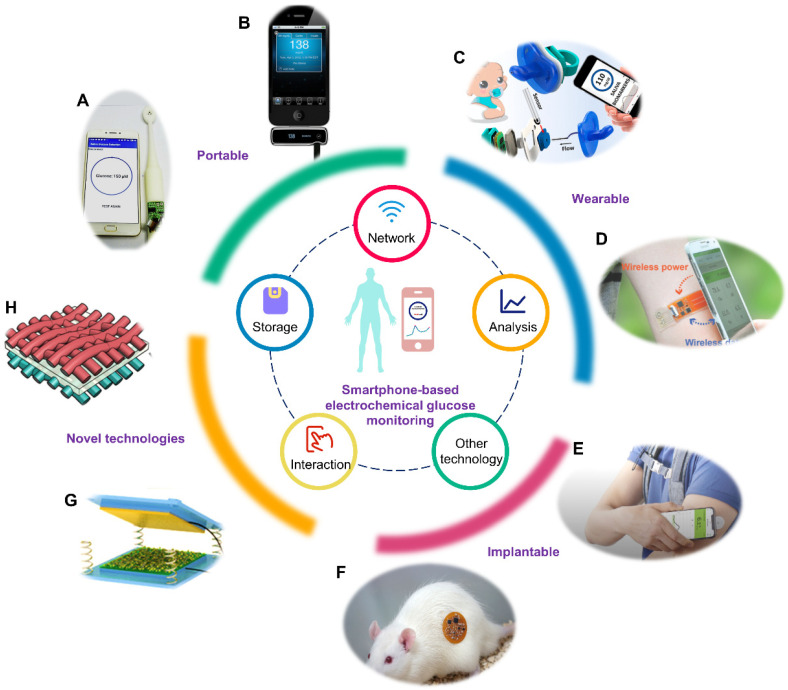
Smartphone-based electrochemical glucose monitoring system. The inner circle represents the technologies integrated in the smartphone. The outer circle represents the realization formats of smartphone-based electrochemical glucose monitoring and novel technologies utilized in this field. (**A**) Portable smart toothbrush for glucose detection in saliva. Reprinted with permission from Ref. [38] Copyright (2019) Elsevier. (**B**) Portable blood glucose device named iBGStar. Adapted from Ref. [39] Copyright. Medgadget, Inc. (**C**) Wearable saliva glucose monitoring with smartphone-based electrochemical system. Reprinted with permission from Ref. [40]. Copyright (2019) American Chemical Society. (**D**) Wearable sweat glucose analysis with battery-free, wireless and flexible electrochemical platform. Reprinted with permission from Ref. [20]. Copyright (2019) Wiley. (**E**) Implantable Free Style Libre Flash Glucose Monitoring System based on interstitial fluid produced by Abbott. Adapted from Ref. [41] Copyright Abbott Laboratories. (**F**) Implantable microelectrode for peritoneal glucose monitoring. Reprinted with permission from Ref. [42] Copyright (2021) Elsevier. (**G**) Structure of a triboelectric nanogenerator. Reprinted with permission from Ref. [43] Copyright (2019) Wiley. (**H**) Schematic diagram of a flexible asymmetric supercapacitor. Reprinted with permission from Ref. [44] Copyright (2020) American Chemical Society.

Herein, based on glucose concentrations under physiological and pathological conditions, the latest advances in smartphone-based point-of-care electrochemical glucose sensing in different biofluids are reviewed in Table 1. As shown in Figure 1, we introduce the technologies integrated in the smartphone which play important roles in the realization of electrochemical testing, including wireless networks, data analysis, storage, interaction with users and other technologies. The subsequent sections encompass the progress of smartphone-based electrochemical glucose monitoring systems in portable, wearable and implantable fields, respectively. Some novel technologies for glucose monitoring systems are also introduced. In each section, the basic configurations, active materials and the function of the smartphone in these sensing devices are illustrated sequentially. In the conclusion, we also discuss the unmet challenges to motivate further technological developments towards smartphone-based glucose monitoring in disease management.

**Table 1:** Summary of glucose concentrations under physiological and pathological conditions.

Biofluid	Physiological Range (mM)	Pathological Range (mM)
Blood	3.9–6.1 [26]	2–40 [27]
Sweat	0.02–0.6 [28]	0.01–1 [29]
Saliva	0.23–0.38 [30]	0.55–1.77 [31]
Tear	0.05–0.5 [32]	0.5–5 [30,33]
Interstitial Fluid	3.9–6.6 [30]	1.99–22.2 [34]
Urine	2.78–5.55 [35]	>5.55 [35]
Ascitic Fluid	Similar to blood glucose [36]	<2.78 [37]

## 2. Capabilities of Smartphones in Electrochemical Monitoring

With the advances and spread of smartphone technology, it can be employed in the glucose detection field as a controller, analyzer, displayer and sharer for rapid, and real-time point-of-care electrochemical glucose monitoring. In this section, we review the technologies integrated in the smartphone which are fully utilized in the electrochemical glucose detection field.

The network of the smartphone enables its communication with electrochemical glucose sensing apparatus [45]. Through the network, the command from the smartphone is transmitted to an electrochemical testing module, and then the testing results of the electrochemical testing module are transmitted to the smartphone. The network system of the smartphone is wired using USB, or wireless by communication protocols such as Bluetooth, near field communication (NFC), infrared, ZigBee, WiFi and ultrawideband [46]. As is shown in Table 2, each type of communication protocol has a different transceiver type, operating frequency, data rate, working ranges, power source and power consumption, with respective availability of suitability for continuous monitoring and sensor network. Among these communication protocols, Bluetooth, NFC and infrared were widely utilized in the electrochemical glucose field. Bluetooth allows multiple devices to exchange information to realize data synchronization over short distances through building a personal area network. The waveband Bluetooth uses is ultrahigh frequency radio waves, from 2.402 GHz to 5 GHz. The main problem with Bluetooth is the information security and the interference between Bluetooth and Wi-Fi. NFC technology, evolved from contactless radio frequency identification, is a short-range radio technology that operates at 13.56 MHz within a distance. Its transmission speed is 106 kb/s, 212 kb/s or 424 kb/s. The reading mode of NFC includes the active mode and passive mode. In the active mode, two NFC terminals, the initiator and target device, can send out a radio frequency field to identify and read information from other NFC devices. In the passive mode, only the initiator generates the radio frequency field and the target device passively responds to it for information transmission [47]. Due to a shorter set-up time and shorter transmission distance, NFC has a higher degree of security than Bluetooth technology. Furthermore, the power is able to be transmitted through NFC, which allows detection devices to harvest power from the smartphone, and furthermore reduces the size of the detection devices [19,20]. Infrared communication protocol is a transmission technology based on infrared rays. In recent years, this technology has made great progress and has been widely utilized in the remote controls of household appliances. As a transmission mode of the wireless local area network, the greatest advantage of infrared is its robustness against radio interferences. However, the transmission is limited because of the short transmission range of infrared ray (less than 5 cm) and its poor transmittance to non-transparent objects [45].

After collecting the raw detection data from a glucose sensor, the smartphone analyzes the data, calculates the value and makes a diagnosis. The advanced data analysis capability of smartphones enables the implementation of glucose detection and disease monitoring easily and powerfully [48,49]. Equipped with high-performance central processing units such as Apple A-Series and Qualcomm Snapdragons, smartphones can perform sophisticated processing, comparable to laptops or even computers [50,51]. Moreover, the inbuilt codes of smartphone application, especially some great algorithms, such as artificial neural networks and machine learning, facilitate the realization of the over-arching goal by identifying certain recurrent patterns [52,53].

In addition, other capabilities of smartphones are utilized in the field of electrochemical glucose detection. With the cooperation of the memory module and the network module, the processed data can be stored in the smartphone or the cloud system, and shared with clinicians through the internet for further analysis and diagnosis [19]. Furthermore, the input button and screen display of a smartphone can provide an interface for interaction between users and glucose detection devices [54].

Briefly, with multiple functions, smartphones efficiently simplify electronic design, minimize volume size, lower the overall cost of the systems and enhance user experience to convenient and real-time glucose monitoring outside of laboratories. Recent progress in smartphone technology could reinforce these strengths and provide more innovative opportunities for electrochemical glucose monitoring.

**Table 2:** Comparison of wireless data communication technologies integrated in smartphones.

Technology Names	Transceiver Type	Operating Frequency	Data Rate	Working Ranges	Power Consumption	Power Source	Continuous Monitoring	Sensor Network	Ref.
Bluetooth	Active radio	2.4~5 GHz	<24 Mbps	10~100 m	Medium	Battery	Yes	Yes	[55,56,57,58,59]
NFC	Passive transponder	13.56 MHz	<424 Kbps	<5 cm	Very Low	Battery-free	No	No	[60,61,62]
Zigbee	Active radio	868 MHz, 915 MHz, 2.4 GHz	250 Kbps	10~100 m	Low	Battery	Yes	Yes	[63,64,65]
Infrared	Active radio	330~350 THz	0.1~1 Mbps	<10 m	Medium	Battery	Yes	No	[66,67]
WiFi	Active radio	2.4~5 GHz	54 Mbps	10~100 m	High	Battery	Yes	Yes	[68,69]
Ultrawideband	Active radio	3.1~10.6 GHz	<480 Mbps	<10 m	Medium	Battery	Yes	Yes	[70,71,72]

## 3. Smartphone-Based Portable Glucose Monitoring

The construction of a smartphone-based portable glucose monitoring system needs the advances in multiple fields. Firstly, the fabrication of a miniaturized glucose sensor requires the combination of technologies in related fields, such as biology, material science, analytical chemistry and mechanical technology [73]. Secondly, the design of the electrochemical testing circuit is to simplify the circuit integrated in the bulky electrochemical workstation, just leaving the module for the specific electrochemical glucose electrode [74,75,76,77]. In addition, smartphones are used to control the electrochemical test, analyze the results, and display and share the testing results, reducing the cost and volume of portable devices. From the perspective of electrode fabrication, electrochemical circuit design and the function of smartphones, we summarize smartphone-based portable glucose monitoring in blood, urine, and saliva samples, respectively. Table 3 lists all smartphone-based portable glucose monitoring systems in this section.

### 3.1. Portable Blood Glucose Monitoring

**Figure 2:**
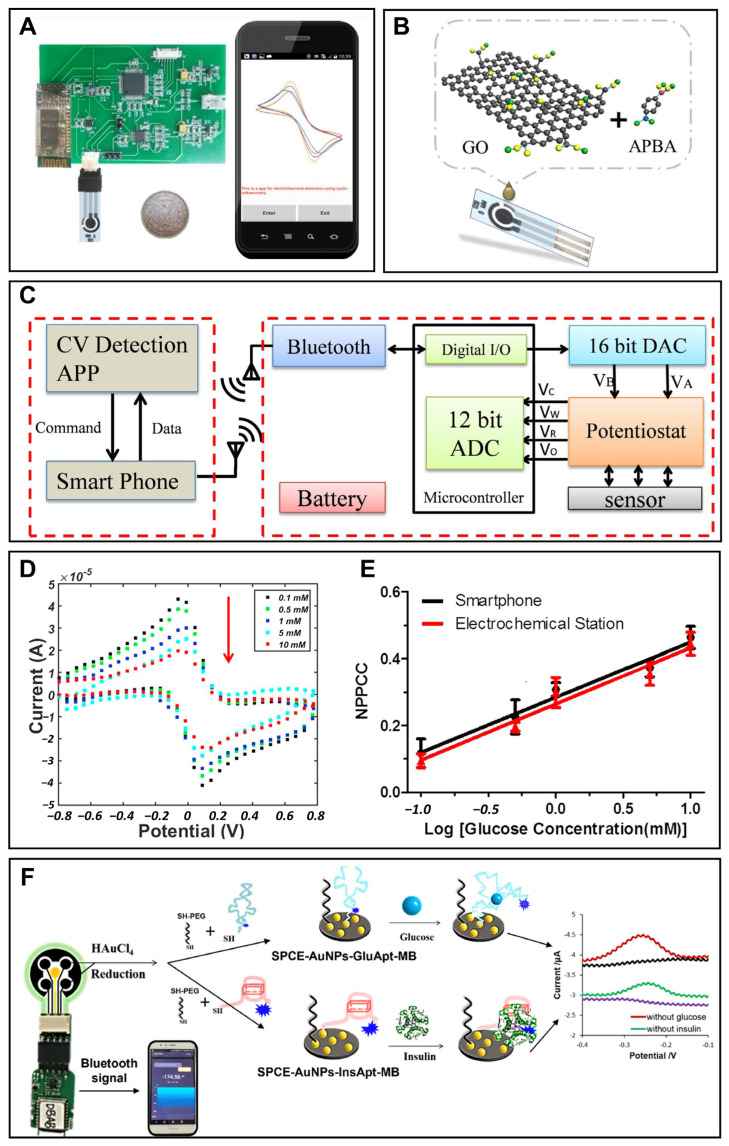
Smartphone-based portable glucose monitoring. (**A**) A smartphone-based glucose detection system with a modified glucose electrode, a portable electrochemical circuit board and a smartphone. Reprinted with permission from Ref. [77] Copyright (2017) Elsevier. (**B**) Schematic diagram showing the modification of the glucose electrode. Reprinted with permission from Ref. [77] Copyright (2017) Elsevier. (**C**) Schematic diagram of the smartphone-based glucose monitoring system. Reprinted with permission from Ref. [77] Copyright (2017) Elsevier. (**D**) Cyclic voltammetry response of the system for glucose of different concentrations. Reprinted with permission from Ref. [77] Copyright (2017) Elsevier. (**E**) Comparison between the calibration curves of the smartphone-based system (black circular) and the commercial electrochemical workstation-based system (red triangle). Reprinted with permission from Ref. [77] Copyright (2017) Elsevier. (**F**) Schematic of a smartphone-assisted electrochemical aptasensor for simultaneous detection of glucose and insulin on a portable biochip. Reprinted with permission from Ref. [82] Copyright (2022) Elsevier.

Because glucose level in blood is one of the most reliable indicators for diabetes monitoring, many studies focus on smartphone-based portable blood glucose monitoring in order to provide convenient, real-time and credible data for diabetes patients and clinics [2]. Ji et al. constructed a smartphone-based electrochemical system for sensitive detection of glucose using the cyclic voltammetric method [77]. The system included a smartphone, a portable electrochemical circuit board, and a functional material modified glucose electrode (Figure 2A,B). A smartphone was used to control the electrochemical module and display the detection result. Consisting of a potentiostat module, an energy transformation module, and a Bluetooth module, the portable electrochemical circuit detector was able to realize the electrochemical cyclic voltammetry test (Figure 2C). The glucose electrode was a screen-printed electrode modified with 3-amino phenylboronic acid and reduced graphene oxide. 3-amino phenylboronic acid was the sensitive substance which was able to combine with glucose molecules and which deterred electron transfer between the solution and the electrode. As shown in Figure 2D, when the glucose level increased, more glucose molecules combined with 3-amino phenylboronic acid on the electrode and then the currents decreased. Generated with the method of reducing the graphene oxide, reduced graphene oxide was utilized to improve the conductivity of the electrode and enhance the sensitivity of the glucose electrode. Integrated with these three components, the smartphone-based glucose sensing system realized glucose detection in the range of 0.1–10 mM, with the limit of detection at 0.026 mM [77]. The calibration curve showed high similarity to the contrast curve from a commercial electrochemical workstation in the same conditions (Figure 2E). Compared with the commercial glucometer, the error was only 3.2%, endorsing the accuracy of this system. Because of the cost-effective property, paper-based electrodes have been widely used to analyze ions, metabolites, proteins and nucleic acids. Therefore, a combination of paper-based equipment and smartphones has also become a trend in the glucose detection field. A smartphone-based electrochemical glucose sensing system with a paper-based electrode was developed for glucose measurement in phosphate-buffered saline [78]. When fabricating a paper-based electrode, a working electrode, reference electrode and counter electrode were directly drawn on the chromatography paper using the carbon pencil. Furthermore, glucose oxidase and potassium ferricyanide solution were dropped on the hydrophilic working electrode area. With the assistance of a miniaturized electrochemical circuit, the paper electrode realized glucose detection in the range of 0–10 mm. The detection data were transmitted through an audio jack to a smartphone and displayed on the smartphone screen. Furthermore, with dual enzymatic reaction channel technology, the smartphone-based electrochemical testing system realized the simultaneous measurement of blood glucose and blood ketone by one whole blood drop from a fingertip, which provided opportunities for clinical identification of diabetic ketosis and diabetic ketoacidosis on the basis of glucose monitoring [79]. In this research, two channels were designed on one proposed test strip, fulfilling the rigid demand for diabetic patients with diabetic ketosis/diabetic ketoacidosis without double pricking the finger to determine the blood glucose and blood ketone. A smartphone was used to control the electrochemical test in two channels, and analyze and display the result on the screen. Furthermore, the accuracy of this smartphone-based dual-channel electrochemical monitoring system was verified by the clinical test with good consistency. A commercial smartphone-based portable blood glucose monitoring system named iBGStar has been created by Sanofi and AgaMatrix, and cleared by the FDA in 2011 [51]. It was the first blood glucose meter that was assisted by an iPhone or iPod Touch. The device automatically realizes the data interaction with the iPhone or iPod Touch with an iOS Diabetes Manager app, and provides users with direct access to data on their mobile device. Through simply tapping the share icon, the users can share data to their health professionals, family, or friends.

### 3.2. Portable Urine Glucose Monitoring

Blood glucose level, which is higher than the renal glucose threshold (180–200 mg/dL), may induce the existence of glucose in urine [6,83]. Furthermore, when the diabetes results in diabetic kidney disease, renal tubules are injured and more blood glucose exists in urine [84]. Therefore, the measurement method of urine glucose was developed, and some smartphone-based portable urine glucose monitoring systems were produced for acquiring more disease information from diabetes patients. Due to the low glucose level in urine, high sensitivity and a low detection range are needed in this field. In this study, gold-copper oxide nanocomposite was synthesized through the co-precipitation method and modified on a glassy carbon electrode [80]. The glucose electrode enabled the amperometric testing of glucose with a wide linear range, fast current response time, and excellent low detection limit. It was established that the modified electrode had high sensitivity, excellent selectivity and good long-term stability. With a screen-printed electrochemical sensor and smartphone technology, the form of urine glucose monitoring was more convenient and could be performed in real-time. Janmee et al. constructed a smartphone-based screen-printed electrode modified with nanocomposite for a single-step measurement of glucose in human urine and electrolyte drinks [81]. Nanocomposite of copper oxide nanoparticles, ionic liquid and reduced graphene oxide improved the properties of the electrochemical glucose sensor. The design of the disposable electrochemical sensor and automated sample pretreatment paper-based device was used to avoid an additional preparation step of urine and electrolyte drinks. In this study, a portable smartphone potentiostat was used to conduct the electrochemical test, and its operation was controlled via the Android application named PStouch, installed in advance. The developed system was successfully applied to the glucose detection in human urine and electrolyte drinks, and the results were correlated with a commercial enzymatic glucose biosensor and labeled values of the commercial products. This system had the advantages of single-step sample loading, low cost, portability, disposability and real-time analysis, and could be an alternative device for a non-enzymatic glucose measurement in urine and drinks.

### 3.3. Portable Saliva Glucose Monitoring

Saliva is massively and continuously produced in the oral cavity by salivary glands. When sampling saliva, the body does not need to be entered by puncture or incision. Therefore, glucose monitoring in saliva can be executed in a non-invasive and hassle-free way [30]. Indeed, some studies have demonstrated the possible correlation between glucose levels in blood and saliva [85]. Thus, smartphone-based portable saliva glucose monitoring was developed by many investigators in recent years. A smart toothbrush was constructed by researchers to detect glucose levels in saliva in real time with the assistance of a smartphone [38]. The authors proved that tin bronze was a suitable sensing material for electrochemical glucose monitoring in saliva. With this material, the smart toothbrush realized glucose detection between 0 and 320 μM, with a sensitivity of 480 μA·mM^−1^·cm^−2^ and a detection limit of 6.6 μM in artificial saliva. The electrode was also highly selective in the presence of ascorbic acid and uric acid. Moreover, tin bronze ensured the robustness of the glucose electrode, which could be regenerated simply by mechanical polishing for long-term use. According to the results in this paper, authors investigated the correlation between responses of the smart toothbrush and glucose concentration in human saliva, and the correlation coefficient of 0.977 endorsed the accuracy of this smart toothbrush in real samples.

When this smartphone-based system was used in practical testing, a potential of −2.0 V for 20 s was first applied on the electrode surface to create a temporary alkaline condition. Then, glucose was detected using the cyclic voltammetric method from 0.45 to 1.0 V at a scan rate of 100 mV/s, with Ag/AgCl as a reference electrode and Pt as a counter electrode. The detection data were transmitted from the smart toothbrush via Bluetooth to the smartphone in real time.

In order to acquire more disease information using a single smartphone-based detection system, some researchers have been trying to construct systems which enable the simultaneous detection of glucose in saliva and other diabetes-related indicators. Liu et al. established a smartphone-based portable biochip to simultaneously monitor glucose and insulin for precise diagnosis of prediabetes/diabetes (Figure 2F) [82]. In this study, the realization of continuous real-time detection of glucose and insulin in saliva demonstrated its possibility of becoming a precise and real-time monitoring tool for diabetes management.

In short, with the assistance of smartphones, portable blood glucose monitoring systems have become more miniaturized and convenient, with significant application value in clinical practice. Due to non-invasive sampling, some interesting and convenient monitoring systems for glucose detection in urine and saliva are demonstrated. Nevertheless, these portable methods have respective limitations. Although blood has been considered as the most thoroughly investigated and understood biofluid for diabetes monitoring, frequent blood sampling could induce physical and psychological burden. The value of urine glucose for diabetes monitoring is limited because the glucose level in urine is influenced by blood glucose level and renal function simultaneously. It is uncertain whether a high urine glucose level indicates high blood glucose concentration or impaired renal function. Hence, in order to evaluate health conditions accurately, urine glucose monitoring should be used in conjunction with other diagnostic methods. The clinical value of saliva glucose level is still uncertain because of the debatable correlation between saliva glucose and blood glucose. Some studies concluded that there was no correlation between salivary and blood glucose concentrations in diabetic patients, whereas other studies demonstrated high correlation between them in the case of diabetic patients [78,80]. Therefore, more reasonable investigations are still needed to clarify the clinical value of salivary glucose detection.

## 4. Smartphone-Based Wearable Glucose Monitoring

Although the portable system reduces the volume of the glucose monitoring device, there is still room to improve the convenience because the collection and pretreatment of the samples are still complex and time-consuming. With the development of flexible materials, one of the proposed solutions was wearable technologies which are capable of continuous, comfortable and in situ monitoring in a non-invasive way. Flexible materials provide stretchable, adhesive, biocompatible and permeable matching mediums between biofluids and the monitoring system, making the monitoring process more convenient and comfortable [86]. Specifically, some flexile microfluidic patches fabricated in some studies improved the efficiency of glucose detection in biofluids [87,88]. Furthermore, flexible electronics enables the electrochemical detection circuit that executes electrochemical detection and records detection data to become wearable [89]. Herein, recent advances towards smartphone-based wearable electrochemical glucose monitoring in sweat, tear, saliva and interstitial fluid were covered and discussed. Wearable glucose monitoring systems in this section are listed in Table 4.

### 4.1. Wearable Sweat Glucose Monitoring

**Figure 3:**
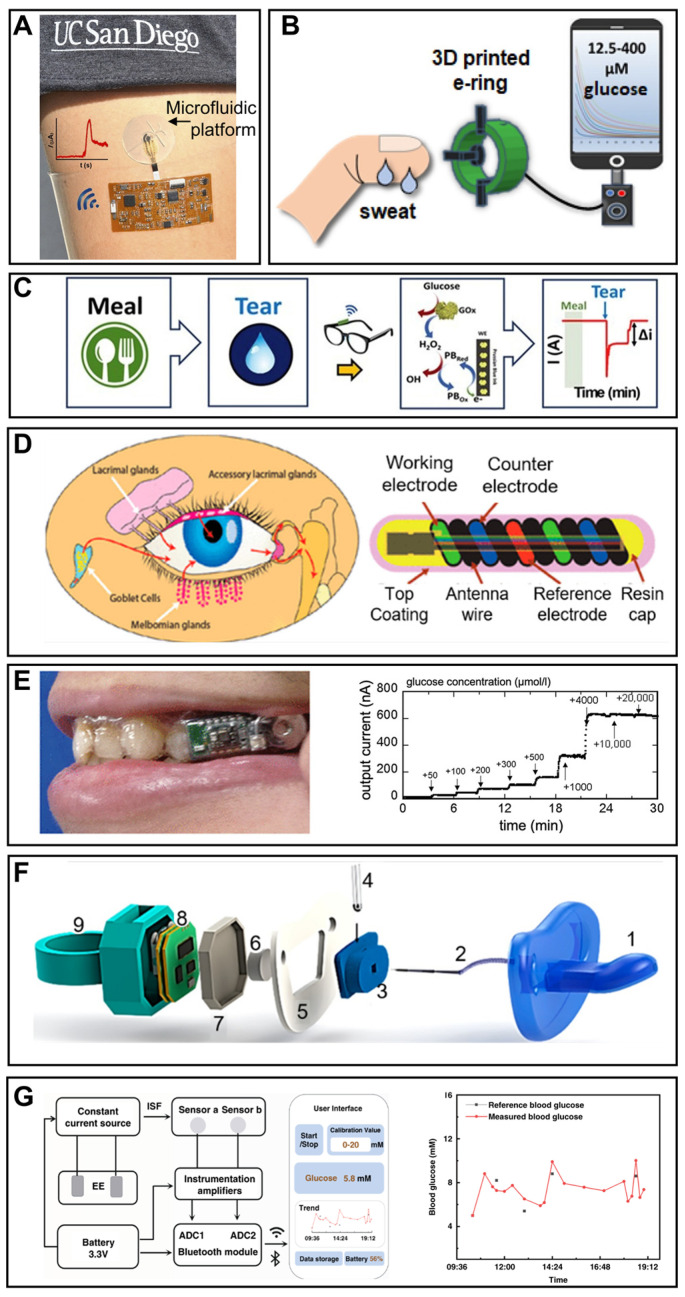
Smartphone-based wearable glucose monitoring. (**A**) In situ sweat glucose analysis based on the skin-mounted wireless electronics equipped with the microfluidic platform. Reprinted with permission from Ref. [90]. Copyright (2017) American Chemical Society. (**B**) Schematic illustration of the detection process of glucose concentration by the 3D-printed e-ring. Reprinted with permission from Ref. [92] Copyright (2020) American Chemical Society. (**C**) The on-body glucose sensing experiments of the eye glasses-based tears biosensor; the glucose levels were recorded for 3 h after a meal. Reprinted with permission from Ref. [100] Copyright (2019) Elsevier. (**D**) The design and structure schema of the NovioSense tear glucose sensor and the production of tear fluid. Adapted from Ref. [94] Copyright (2018) American Chemical Society. (**E**) Photograph of the mouthguard glucose sensor with wireless module and battery for salivary glucose monitoring and its responses to different glucose concentrations. Reprinted with permission from Ref. [97] Copyright (2020) American Chemical Society. (**F**) Scheme of the assembling of the pacifier and its matching medium: (1) nipple of the pacifier, (2) inlet for collecting saliva, (3) electrode chamber, (4) glucose electrode, (5) central piece, (6) outlet, (7) insulator pacifier cap, (8) integrated wireless potentiostat, and (9) back cap of the pacifier. Reprinted with permission from Ref. [40] Copyright (2019) American Chemical Society. (**G**) The systemic block diagram of a smartphone-based interstitial fluid glucose monitoring watch and blood glucose variation curve of a volunteer measured by this system. Adapted from Ref. [99] Copyright (2022) Springer Nature.

As the most readily obtainable biofluid from sweat glands distributed all over the body, sweat provides accessible sampling sites. In order to realize wearable sweat glucose analysis, the relatively lower concentration than blood and the difficulties in obtaining a sufficient amount of sample collection need to be considered fully. Sweat generation is usually accomplished through exercise or iontophoretic stimulation. Aida et al. developed a skin-mounted microanalytical sweat metabolites monitoring system for continuous real-time monitoring of lactate and glucose in human sweat with the help of epidermal technology (Figure 3A) [90]. The glucose sensor was fabricated through screen-printing. A reference electrode was printed using silver silver/chloride ink. Then, glucose oxidase was mixed with carbon Prussian blue ink and used to print the working electrode. This screen-printed flexible senor enabled glucose monitoring in sweat from 2 to 10 mM with a limit of detection of 50 μM. Considering the matching mediums between sweat and the glucose sensor, this study designed a microfluidic platform. In simulation testing, this microfluidic platform sped up sweat flow rate and shortened the device filling time. Meanwhile, it endured repetitive mechanical deformations experienced by the epidermis. The wearable system was successfully applied to monitor glucose and lactate levels during cycling activity of different healthy subjects. Most wireless smartphone-based sensors usually need on-board batteries, which often restrain the miniaturization of the system. Xu et al. proposed a NFC technology-supported wireless epidermal electrochemical system as a battery-free design for sweat biomarker detection including glucose [20]. The fully integrated system could realize wireless energy harvesting and data transmission with smartphones. Indeed, successful on-body monitoring of glucose, H^+^, Na^+^ and K^+^ was fulfilled by this battery-free and wireless system. However, the immobilized enzymes could induce some critical problems. Specifically, the activity of enzymes could be affected by environmental factors that cannot be well controlled under in situ monitoring. The degradation of enzymes could also affect the shelf life and the long-term monitoring possibility of sensors. Moreover, the immobilization of enzyme on the electrode might decrease the activity of the enzyme and slow down the electron transfer. Herein, Zhu et al. proposed a fully integrated wristband equipped with a nonenzymatic wearable sensor for continuous electrochemical analysis and real-time glucose monitoring. The system could realize a linearity response correlated to glucose concentration from 30 to 1100 μM. This wristband monitored the perspiration glucose during physical activity, and uploaded the test results to a smartphone application via Bluetooth [91]. Katseli et al. combined optical and electrochemical techniques and fabricated a single-step 3D-printed e-ring as a solution to wearable noninvasive glucose detection in sweat (Figure 3B) [92]. The e-ring could resist mechanical bending and enabled detection in the physiological concentration range of glucose within 12.5–400 μM. Moreover, the e-ring was shown to successfully track the glucose level variation before and after a meal.

### 4.2. Wearable Tear Glucose Monitoring

Tears, also known as lachrymal fluid, are from the secretion of the lachrymal gland covering the eye. Han et al. proposed a noninvasive preocular sensor system based on the commercially available electrochemical sensor and dry eye syndrome (a prevalent complication of diabetes) sensor for the continuous monitoring of diabetes and dry eye syndrome [93]. It could estimate the blood glucose levels by the result acquired from the system with acceptable accuracy that was in validation with that of a clinical glucometer. Sempionatto et al. fabricated an eyeglasses-based tears biosensor consisting of a microfluidic electrochemical detector (Figure 3C) [100]. The tear-sensing platform could avoid the direct contact of sensor systems with the eye and realize key tear biomarker (alcohol, glucose and vitamin) monitoring. Kownacka et al. demonstrated a NovioSense minimally invasive tear glucose sensor for the measurement of glucose in the tear of type 1 diabetes patients [94]. The flexible design and polysaccharide hydrophilic coating induced a non-immune response in the eye, and the sensor realized the accurate recording of glucose levels in a clinical test (Figure 3D). At present, contact lenses have been a considerable platform to integrate with miniaturized glucose sensors, and have attracted more attention than other kinds of wearable health care devices for tear glucose detection because of the preferable outlook that serves as the window to the body. Keum et al. proposed a smart contact lens based on a microcontroller chip and ultrathin flexible electrical circuits built on biocompatible polymer [95]. The device was capable of various functions including real-time electrochemical biosensing, on-demand drug delivery, wireless power management, and data communication. The noninvasive and successive diabetic diagnosis and retinopathy therapy were demonstrated using the smart contact lens. A long-term robust continuous glucose monitoring of a smart contact lens was reported [101]. Authors successfully constructed smart contact lenses containing gold-platinum bimetallic nanocatalyst modified with hyaluronate in nanoporous hydrogels. This smart lens could accurately measure blood glucose levels with high sensitivity, a low detection limit, low hysteresis, and a fast warming-up time. In diabetic rabbits, this system reflected rapidly changing blood glucose levels very sensitively. Additionally, it could measure the tear glucose levels in a human with data transmission to a smartphone.

### 4.3. Wearable Saliva Glucose Monitoring

Secreted by the capillaries of salivary glands, saliva contains a wide range of biomarkers, such as glucose, lactate, hormones, enzymes and antibodies, and thus is becoming a focus of wearable healthy monitoring [102]. The construction of wearable saliva glucose monitoring needs to consider the lower glucose level compared with blood glucose, full biocompatibility of the system for intraoral settlement, the high concentration of interference from other electroactive matters, and the inevitable biofouling induced from the high protein content and bacteria. Arakawa et al. developed a detachable “cavitas sensor” by incorporating Pt and Ag/AgCl electrodes modified by an enzyme membrane on a mouthguard for glucose monitoring in a human oral cavity [96]. The glucose sensor could sensitively detect glucose over 5–1000 μM, which includes the range of human saliva glucose from 20 to 200 μM. Moreover, 5-h in situ stable monitoring was achieved by the telemetry system. However, the interference of ascorbic acid and uric acid that is rich in saliva was still a problem for the precise monitoring of glucose in the oral cavity. Thus, the same research group investigated an interference rejection membrane to improve the selectivity of the glucose sensor [97]. The device successfully suppressed the influence of ascorbic acid and uric acid during continuous salivary glucose monitoring. Furthermore, this mouthguard glucose sensor equipped with a cellulose acetate membrane could detect glucose concentration from 1.75 to 10000 μM in artificial saliva, and realized the glucose analysis in a saliva sample (Figure 3E). A glucose monitoring pacifier was demonstrated for infants [40]. The glucose sensor was fabricated via screen-printing three-electrode configuration using different ink. The modification of a working electrode consisted of dropping the chitosan layer, drying it at room temperature, dropping the glucose oxidase layer and drying it again at 4 °C. The matching medium between saliva and glucose was elaborately designed. As shown in Figure 3F, a glucose sensor was inserted into a chamber. Authors utilized natural mouth movements of infants to promote saliva flow from the mouth to the sensor chamber and designed a channel to make sure that the flow was unidirectional. Finally, a hydrophilic thread which connected the electrochemical chamber to the outside of the pacifier was used to update the sample continuously. Thus, this initial design of a glucose monitoring pacifier introduces new possibilities for glucose monitoring in infants and neonates.

### 4.4. Wearable Interstitial Glucose Monitoring

Interstitial fluid (ISF) exists around the skin cells to provide nutrients by ways of direct diffusion from the source of capillary endothelia. Unlike other aforementioned biofluids, wearable interstitial glucose monitoring needs non-invasive sampling methods. Reverse iontophoresis has been considered as a solution for ISF withdrawal from the skin surface without harm to the integrity of skin, realized by applying an electric field to drive the ISF through the skin in the form of an electroosmotic flow. Kim et al. developed a wearable epidermal platform for simultaneous noninvasive sampling and analysis of glucose and alcohol in both sweat and ISF [98]. Additionally, the on-body analysis of both biomarkers following meal and drink consumption of healthy human subjects showed validated correlations to commercial blood glucometers and breath-analyzers. Efforts have been made for the realization of clinical practice using ISF-based glucose monitoring. Chang et al. reported a Nafion-coated flexible electrochemical sensor patch integrated on a watchband for the epidermal extraction of ISF at the wrist [99]. The in situ and real-time glucose levels could not only be displayed on the watch, but also checked through the designed smartphone application. As for clinical use, the integrated watch realized 84.34% accuracy in 23 volunteers, including 12 diabetic patients and 10 healthy people (Figure 3G).

Smartphone-based wearable electrochemical glucose monitoring systems have developed rapidly and become the focus of immense research activities. Extensive efforts have led to innovative and interesting progress in this field. The emergence of noninvasive, continuous and comfortable monitoring methods provides the chance of improving the life quality of diabetic patients. However, these wearable monitoring methods still face a lot of challenges for clinical application. For wearable glucose monitoring in sweat, tears and saliva, the correlation between glucose level in blood and these biofluids are debatable, resulting in an uncertain practical application value. In addition, these compositions of biofluids secreted on the body surface can be easily influenced by the immediate environment. Changes of wind, hydration and physical activities could induce physiological changes of these biofluids, leading to varied glucose levels within them. Although ISF glucose analysis is valuable for diabetes monitoring, the realization of this analysis method necessitates reverse iontophoresis technology, which still faces some challenges. For example, this technology might induce side effects such as pain and irritation, which was proved to be one of reasons for the withdrawal of the first commercial reverse iontophoresis-based device [103]. In addition, the potential sample contamination and dilution from reverse iontophoresis also limits the practical application of this method.

## 5. Smartphone-Based Implantable Glucose Monitoring

In the electrochemical field, due to certain significance of blood and ISF glucose analysis to diabetes management, blood-based and ISF-based implantable glucose monitoring still need to be developed. In recent years, with the developments of nanomaterials, micromachining and antifouling technologies, the implantable electrochemical electrode is becoming minimally invasive, highly sensitive and more selective, providing an opportunity for realizing comfortable and accurate glucose monitoring. Based on the list of implantable glucose monitoring systems (Table 5), we consecutively summarize implantable glucose monitoring systems in blood and other samples.

### 5.1. Implantable Blood Glucose Monitoring

Implantable blood glucose in situ monitoring faces a great challenge because of the more complex components and the greater interference in blood. A smaller size was also required in blood implantation because large electrodes posed a risk of bleeding. In 1998, Schmidtke et al. realized glucose monitoring in 4 h in jugular veins [108]. However, the diameter of the glucose sensor was about 300 μm, causing a large invasion to jugular veins. The implantation progress required a cut in the vein and the help of a silicone tube in advance. Hence, there was still much room for decreasing the size of the glucose electrode, simplifying the implantation process and reducing the invasion. Wang et al. constructed a blood glucose sensor in situ based on carbon nanotube fiber bundles [104]. Being lightweight, the fiber bundles mimicked the structure of muscle and exhibited an outstanding mechanical match and great compatibility with biological tissues. The diameter of fiber bundles ranged from one to hundreds of micrometers. For glucose sensing, zinc oxide, multi-walled carbon nanotubes, and Nafion was modified on the fiber bundles. Researchers implanted the modified fiber bundles in the femoral vein of a cat for continuous blood glucose monitoring. The results showed that zinc oxide-based glucose fiber bundles remained stable and effective over continuous testing in vitro and monitoring in vivo for 28 days. A flexible patch in contact with the fiber bundles was worn on the skin of the cat, and it could transmit the detection data via Bluetooth. A smartphone application was designed to accompany the integrated system and provide a user interface for data collection. With minimal invasion and great biological performance, this smartphone-based implantable glucose monitoring system provided a potential solution for long-term blood glucose monitoring in situ.

### 5.2. Implantable Glucose Monitoring in Other Biofluids

**Figure 4:**
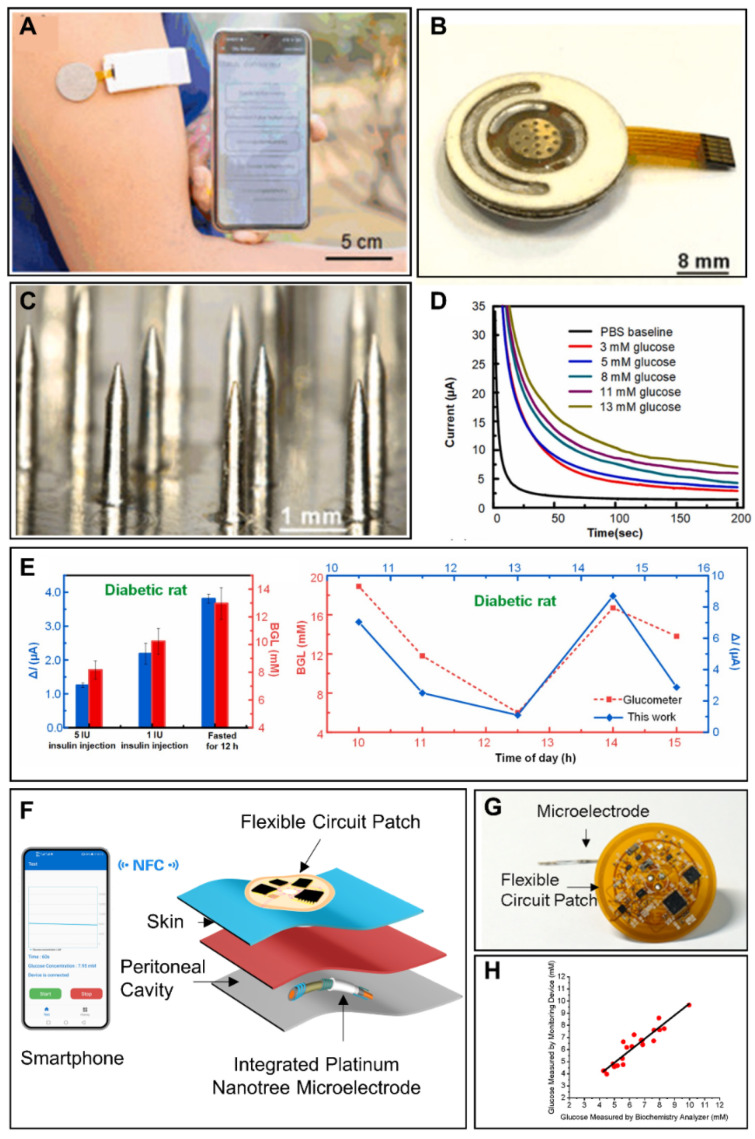
Smartphone-based implantable glucose monitoring. (**A**) Smartphone-based ISF glucose analysis with microneedle array. Reprinted with permission from Ref. [106] Copyright (2022) Elsevier. (**B**) Photograph of an ISF glucose sensor. Reprinted with permission from Ref. [106] Copyright (2022) Elsevier. (**C**) Optical photo of a microneedle array. Reprinted with permission from Ref. [106] Copyright (2022) Elsevier. (**D**) The chronoamperometric responses of a glucose sensor under subdermal glucose concentrations of 3–13 mM. Reprinted with permission from Ref. [106] Copyright (2022) Elsevier. (**E**) In vivo glucose sensing performance of the smartphone-based ISF glucose analysis system in diabetic rats. Reprinted with permission from Ref. [106] Copyright (2022) Elsevier. (**F**) Schematic diagram of implantable ascitic glucose monitoring system. Reprinted with permission from Ref. [42] Copyright (2021) Elsevier. (**G**) Optical image of integrated platinum nanotree microelectrode and a flexible circuit patch. Reprinted with permission from Ref. [42] Copyright (2021) Elsevier. (**H**) Comparison between glucose concentrations measured by a smartphone-based ascitic glucose monitoring system and a biochemical analyzer. Reprinted with permission from Ref. [42] Copyright (2021) Elsevier.

At present, ISF-based glucose detection is the widest research field among implantable glucose detection approaches. The main commercial forms among implantable glucose monitoring methods are ISF-based electrochemical devices. The main reason for this is that the significance of ISF glucose concentration on blood glucose and diabetes has been confirmed. Although glucose transport resistance exists between capillary blood and the subcutaneous fluid induces the transient difference of two results, this difference is able to be corrected through some models [108,109]. In addition, the realization of ISF glucose detection is easy and less invasive compared with blood glucose monitoring. In the smartphone-based implantable electrochemical glucose monitoring field, a single needle-type glucose sensor is a common form for glucose detection in ISF. Lei et al. developed a nonenzymatic electrochemical sensor for implantable glucose monitoring in ISF [110]. The needle-type glucose sensor was a gold wire modified by Nafion, tetrahydrofuran and polyurethane. The glucose sensor was able to be implanted under the skin for glucose detection in ISF for 30 days. A smartphone in this study was used to wirelessly communicate with an implantable system, analyze the data and display the results with customized application. The Abbott company developed a commercial continuous detection system for ISF glucose monitoring. The Free Style Libre Flash Glucose Monitoring System mainly consisted of an NFC-enabled smartphone (or a detector) and an implantable needle-type glucose sensor with a length of 5 mm and a width of 0.45 mm [111]. The needle-type glucose sensor could be implanted under the skin of the patient’s upper arm through a spring-loaded inserter and then kept available for 14 days. When glucose concentration needed to be detected, the user put the smartphone (or the detector) close to the glucose sensor and the glucose concentration in ISF was detected and the data were transmitted through NFC to the smartphone (or the detector). The system did not require external calibration with finger-prick blood glucose detection. Test results from 72 participants demonstrated that the device had enough accuracy compared with blood glucose detection [105]. Recently, microneedle technology has been another attractive field to realize glucose detection in ISF because of the minimal invasion. The needles are short in length, which enables the microneedles to puncture the stratum corneum with limited damage to nerves and vessels in the dermis [112]. There are two kinds of microneedles for glucose detection: hollow microneedles and solid microneedles [113]. The design of hollow microneedles protects the electrode, thus avoiding damage to the electrode materials and skin tissues. Conversely, solid microneedles have greater mechanical strength and a much simpler fabrication method. Cheng et al. constructed a smartphone-based glucose detection system with a solid microneedle array (Figure 4A,B) [106]. As shown in Figure 4C, the average height, tip diameter, and base diameter of the microneedles were approximately 2.2 mm, 30 μm, and 400 μm, respectively. The microneedle array was modified with glucose oxidase. Furthermore, the authors utilized reverse iontophoresis technology to extract glucose and improve the detection performance of the glucose sensor in ISF. The smartphone in this study was used to control the reverse iontophoresis parameter and execute electrochemical glucose detection. Moreover, the processed data in the microcontroller unit was transmitted to the smartphone via Bluetooth based on the BLE 4.0 protocol stack, real-time displayed in the Android application, and stored in the smartphone. This smartphone-based microneedle array system realized glucose detection between 3–13 mM (Figure 4D). In vivo experiments revealed a high correlation between the results measured by this system and commercial blood glucometers (Figure 4E). The minimal invasion microneedle was also used for detection of multiple targets. Simultaneous detection of glucose, uric acid and cholesterol was realized using a flexible microneedle electrode array-based biosensor and a multi-channel portable electrochemical analyzer [107].

Though peritoneal glucose has important indicating function in peritoneal carcinomatosis and peritonitis monitoring, the glucose monitoring system for ascitic fluid monitoring is still seldom established. Xu et al. developed an implantable platinum nanotree microelectrode with a flexible electrochemical patch for in vivo and real-time peritoneal glucose detection (Figure 4F,G) [42]. Modified with the platinum nanotree, the microelectrode had high sensitivity. The diameter of the microelectrode was about 200 μm, and the microelectrode was implantable in the peritoneal cavity in a minimally invasive way. The flexible circuit patch was used to execute the electrochemical test and realize wireless power harvesting and data interaction with an NFC-enabled smartphone. The whole system was able to detect glucose dynamics in vivo in a rat peritoneal cavity. Furthermore, the accuracy of this system was validated in ascites of patients (Figure 4H). In this way, the system offered hassle-free, rapid and minimally invasive opportunities toward peritoneal glucose monitoring.

Obviously, implantable blood glucose analysis with minimal invasion provides an accurate, hassle-free and comfortable chance for continuous diabetic monitoring. This analysis method faces vital challenges due to the possible bleeding consequences and other interferences. With the efforts of investigators, advances were made recently. Various implantable ISF glucose devices were constructed, along with the progress of single needle-type electrodes and microneedle array technology. Commercial products are already emerging, and some of them have been proven to improve the life quality of diabetic patients. Peritoneal glucose monitoring has significance in the management of some peritoneal diseases and deserves more attention.

## 6. Novel Technologies for Glucose Monitoring

For the comprehensive applications of wearable and implantable glucose biosensing systems, challenges always exist, such as the construction of a stable and wearable energy supply [114]. traditional energy supply solutions, such as batteries, cannot be used for miniaturized wearable devices that can be worn easily. Meanwhile, the limited life and possible pollution issues of the batteries could also hinder the practical deployment of the wearable glucose monitoring systems. NFC technology with the support of a smartphone has been integrated into wearable systems as a promising candidate [19,20]. However, the NFC solution is limited by the distance between the device and the smartphone. This limitation could be much more critical when it comes to implantable devices aiming for long-term and in-body operations. The current approach for this demand is wearable and environmentally friendly energy harvesting technology that could obtain sustainable energy directly from the environment [115,116]. Novel energy sources suitable for glucose monitoring systems include mechanical energy, thermal energy, chemical energy and solar energy. Substantial efforts have been made to combine different forms of energy harvesting systems with smartphone-based electrochemical glucose monitoring systems [117].

### 6.1. Biofuel Cells for Glucose Monitoring

**Figure 5:**
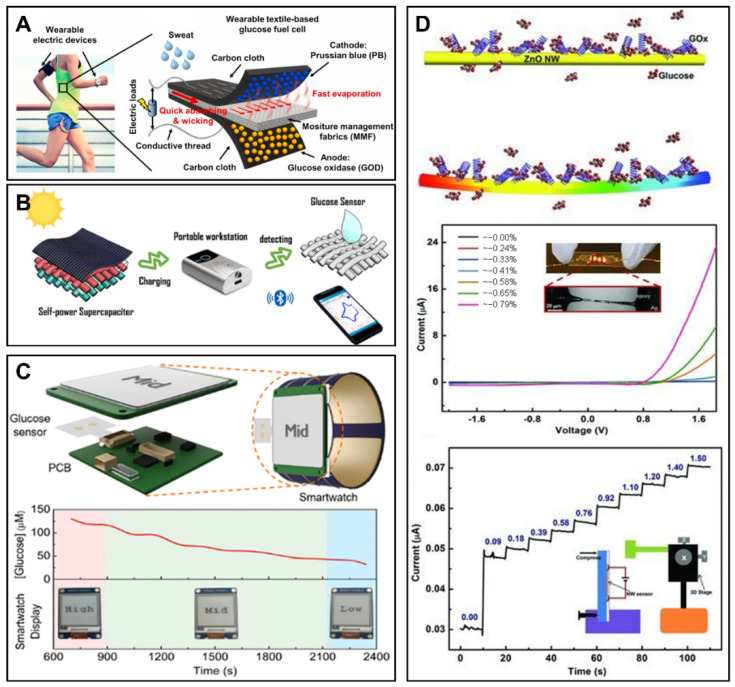
The novel energy supply technology for glucose monitoring. (**A**) The schematic view of the wearable and flexible textile-based glucose biofuel cell based on moisture management fabric. Reprinted with permission from Ref. [120] Copyright (2020) Elsevier. (**B**) The illustration of the wearable energy storage and flexible glucose detection based on self-power supercapacitor. Reprinted with permission from Ref. [44] Copyright (2020) American Chemical Society. (**C**) Schematic illustrations of the integrated and self-powered smartwatch, and the real-time monitoring of sweat glucose. Reprinted with permission from Ref. [125] Copyright (2019) American Chemical Society. (**D**) Illustration of the zinc oxide-based piezotronics for the enhanced self-powered detection of glucose. Reprinted with permission from Ref. [126]. Copyright (2013) Wiley.

A biofuel cell (BFC) is a kind of fuel cell which converts the chemical energy of organic matter into electrical energy with the help of biocatalysts. The main categories of biofuel cells include microbial fuel cells and enzymatic fuel cells. Microbial fuel cells exploit bacterial respiration as a redox reaction where electrons are extracted from bacterial food sources and feed into an electrical circuit to generate power. The advantages of microbial fuel cells are high conversion efficiency and efficient operation at room temperature. The particularity of an enzymatic fuel cell is the replacement of bacteria with redox enzyme catalysts, with a considerable gain in volumetric catalytic activity, but at a substantially higher cost [118]. Jeerapan et al. proposed a biofuel cell based on aa highly stretchable textile for the construction of self-powered devices for the monitoring of glucose and lactate [119]. The textile was screen-printed using carboxylic acid and hydroxyl functionalized multi-walled carbon nanotube-based stress-enduring inks with a single-enzyme and membrane-free design. The linearity of power density with the glucose concentration lay in the range of 0–50 mM, with a sensitivity of 3.14 μW·cm^−2^·mM^−1^ and with a maximum power density of 160 μW cm^−2^ and 0.44 V open circuit voltages [119]. Additionally, the self-generated real-time signals were obtained from the sock-based BFCs and self-powered sensors in a sweat environment with a smartphone and an integrated wireless device. The existence of biofuel depletion and solvent evaporation limited the stability and lifetime of BFCs. Wang et al. demonstrated a wearable and flexible textile-based glucose BFC based on moisture management fabric to form a high-speed, long-term continuous flow, which could provide enough fuel supply and molecule transport between the glucose oxidase immobilized anode and the Prussian blue modified cathode [120]. The multi-stack BFC could ensure 1.08 V of open circuit voltage and 80.2 μW of maximum power, and successfully realized continuous monitoring of sweat glucose during physical exercise (Figure 5A). The simultaneous self-powered sensing of multiple physiological parameters is of great importance for real-time in situ human metabolic health monitoring [121]. The Gao group proposed a battery-free, biofuel-powered electronic skin for multiple tasks including efficient energy harvest, multiplexed biomarker sensing and wireless data transmission. A record-breaking power density of 3.5 mW cm^−2^ was realized, which could support sensing and Bluetooth operation. On-body personalized metabolic monitoring (NH_4_^+^, urea, temperature, glucose, and pH) was realized by the forehead-mounted e-skin system [122].

### 6.2. Supercapacitors for Glucose Monitoring

Owing to their fast charging/discharging properties and long lifetimes, supercapacitors have attracted great attention in the development of wearable electronics. Moreover, the needs of wearable devices, such as flexibility and deformations, can be satisfied by the emerging all-solid-state supercapacitors. Sun et al. proposed a supercapacitor based on electrochemical deposition of carbon fiber and nickel cobalt dioxide nanosheet and nitrogen-doped carbon, and applied it to enzyme-free glucose monitoring (Figure 5B) [44]. The excellent behaviors and electrocatalytic properties of the dual-functional active material helped the supercapacitors exhibit high capacitive stability (94% capacitive retention remaining after 10,000 cycles) and high sensitivity of 592 μA·mM^−1^ for the detection of glucose. After the integration of a Bluetooth module, the health monitoring of glucose in physical practice was realized by this wearable self-powered smart glucose sensor under the control of a smartphone. Based on accounts of diabetes patients, deliberate and unnecessary physical activities for sweat induction are considered burdens. Therefore, Kil et al. integrated a microneedle-based glucose sensor with a self-powered solid-state supercapacitor [123]. This patch-type all-in-one system could realize glucose detection by the glucose oxidase modified polymer microneedle, and the energy was supplied by the electrons from the electrochemical reaction of glucose. The all-in-one system successfully recognized normal, prediabetic and diabetic glucose levels, and could perform consistent self-charging from constant glucose supplementation when attached to human skin.

### 6.3. Other Energy Supply Technologies for Glucose Sensors

Human body temperature is constant, and a temperature difference always exists between the human body and the surrounding environment. Hence, thermoelectric generators can stably convert body heat into electric energy for practical usage uninterruptedly, 24 h a day. Kim et al. demonstrated a wearable thermoelectric generator integrated with a lithium-sulfur battery and a commercial glucose sensor [124]. The continuous power supply of the thermoelectric generator could reach 378 μW, and it could charge the lithium-sulfur battery, providing a stable voltage of 2 V, or operate a commercial glucose sensor of 64 μW. The thermoelectric generator-battery system presented potential applications for continuous long-term health monitoring of glucose and other biomarkers.

Solar energy is a green and renewable clean energy which can be obtained from sunlight endlessly, and has been considered as an ideal power source for self-powered electronic devices. Zhao et al. proposed a fully integrated and self-powered smartwatch for the real time monitoring of sweat glucose [125]. The smartwatch consisted of electrochemical glucose sensors, customized circuits, display units, and flexible photovoltaic cells and rechargeable batteries (Figure 5C). The real-time sweat glucose level profiles during iontophoresis, indoor biking and outdoor running were recorded successfully by the smartwatch, providing a promising protocol for statistical investigation of sweat contents and human dynamics.

Piezoelectricity could be repetitively generated from the mechanical deformation of materials that have piezoelectric property. The mechanical energy can be harvested during human motion for the functioning of wearable glucose monitoring devices. Wang’s group reported a self-powered glucose sensor based on the piezotronic effect of mental-semiconductor-medal structured zinc oxide nanowire (Figure 5D) [126]. The sensitivity of the glucose sensor was improved by over 300% under the enhancement of the piezotronic effect.

With the advantages of being small in size, low-weight, easy to wear and pollution-free, self-powered technologies provide a promising direction for smartphone-based glucose monitoring. In the last decade, self-powered electrochemical glucose detection has made a significant progress. However, most reported self-powered electrochemical sensors are in the proof-of-concept state, and the power efficiency of these devices is still limited in practical application of continuous glucose monitoring. Further efforts are needed to realize the wearable and implantable self-powered glucose monitoring systems.

## 7. Summary

In this review, we summarize the latest progress of smartphone-based electrochemical glucose monitoring systems (Table 6). With the function of wireless sending and receiving, data analysis, displaying and interaction with users, smartphones have been used in the electrochemical glucose monitoring field to simplify the design, reduce the cost and improve user experience. With the assistance of smartphones, portable glucose detection sensing has acquired some special advantages and developed rapidly, entering the stage of commercialization. Wearable glucose monitoring was realized through flexible and comfortable electrochemical glucose detection in ISF, saliva, tear and sweat. In the implantable glucose monitoring field, a single microelectrode and microneedle array was used to reduce the invasion and maintain the reliability of glucose detection. In order to solve the problem of energy supply, especially in wearable and implantable sensing systems, some novel energy sources have been proposed.

## 8. Challenges and Future Directions

Challenges remain in the field of smartphone-based electrochemical glucose monitoring. Although commercial portable glucose monitoring systems have been constructed, there is still room for improving the testing accuracy in order to acquire more reliable data for disease monitoring. To further develop wearable electrochemical glucose monitoring and apply wearable systems to clinical practice, the challenges to be faced include the unclear and disputable correlation between glucose levels in blood and in some biofluids which are secreted on body surface, as well as the requirement of a low detection range and high sensitivity because of the lower glucose concentration in them. As for implantable glucose monitoring, biocompatibility and biosafety are factors to consider. Although various trials on large animals have been conducted, human trials are still needed to confirm the safety of the fully encapsulated system. Less invasion and longer availability are challenges which must be solved when the monitoring system is aimed to be applied in clinical practice.

Recent advances in many fields provide future directions to smartphone-based electrochemical glucose monitoring. For example, advanced nanomaterials develop rapidly in the last decade and offer an attractive opportunity to design highly sensitive sensors for glucose monitoring in alternative biofluids [127]. Some specific nanoscale materials have also been developed with the aim of eliminating interferences in biofluids and enhancing the stability of sensors [128]. Thus, advanced nanomaterials may provide an important direction to realize accurate detection with compact glucose sensors. Secondly, novel power sources are also a promising direction in this field. On the one hand, the practice of self-powered technology is undergoing continuous evolution and efforts are being made in order to build compact self-powered glucose monitoring systems with practical value. On the other hand, novelty battery technology is being developed and batteries are becoming flexible, printable and ultra-thin, all of which are in line with the demand for new generations of wearable and implantable glucose monitoring systems [129,130]. Thirdly, smartphone-based glucose monitoring systems are expected to be part of a fully closed-loop system. Recent progress in smartphone-based artificial intelligence algorithms has improved the efficiency of diagnosis and treatment in different disease management [131,132]. Therefore, novel smartphone algorithms could potentially be used to predict glucose fluctuations, accelerate the decision-making process, and eventually assist the drug delivery system to accurately manage glucose-related diseases [133,134]. Finally, the development of multiplexed assays for disease-related markers would provide a broader understanding of patients’ health than a single glucose-sensing assay, and thus improve disease management.

## Figures and Tables

**Table 3:** List of smartphone-based portable glucose monitoring systems.

Biofluid	Limit of Detection (μM)	Working Range (mM)	Ref.
Blood	26	0.1–10	[77]
Blood	0	0–10	[78]
Blood	-	6.9–23.1	[79]
Blood	-	1.1–33.3	[51]
Urine	1.4	0.005–0.65	[80]
Urine	0.14	0.03–7	[81]
Saliva	6.64	0–0.32	[38]
Saliva	80	0.1–50	[82]

**Table 4:** List of smartphone-based wearable glucose monitoring systems.

Biofluid	Limit of Detection (μM)	Working Range (mM)	Ref.
Sweat	50	2–10	[90]
Sweat	-	0.1–0.5	[20]
Sweat	15	0.03–1.1	[91]
Sweat	1.2	0.0125–0.4	[92]
Tear	0	0–1	[93]
Tear	0	0–20	[94]
Tear	0	0–2.78	[95]
Saliva	-	0.005–1	[96]
Saliva	-	0.00175–10	[97]
Interstitial Fluid	0	0–0.16	[98]
Interstitial Fluid	-	0.02–0.2	[99]

**Table 5:** List of smartphone-based implantable glucose monitoring systems.

Biofluid	Limit of Detection (μM)	Working Range (mM)	Ref.
Blood	50	2.5–7	[104]
Interstitial Fluid	-	2.22–27.78	[105]
Interstitial Fluid	920	3–13	[106]
Interstitial Fluid	260	2–12	[107]
Ascitic Fluid	53.9	1–20	[42]

**Table 6:** Summary of smartphone-based electrochemical systems for glucose monitoring in biofluids.

System	Main Technologies	Convenience	Continuous Detection	Glucose Monitoring in Biofluids		
				Biofluid	Sampling Method	Correlation with Blood Glucose Level
Portable system	Smartphone-related technologies, Miniaturized electrochemical circuit	Medium	No	Blood	Invasive	Equal
				Urine, Saliva	Non-invasive	Debatable
Wearable system	Smartphone-related technologies, Miniaturized electrochemical circuit, Flexible electronics	High	Yes	Sweat, Tear, Saliva	Non-invasive	Debatable
				Interstitial fluid	Non-invasive, Irritation from RI	Correlated
Implantable system	Smartphone-related technologies, Miniaturized electrochemical circuit, Flexible electronics, Biocompatible and antifouling microelectrode	High	Yes	Interstitial fluid	Minimally invasive	Correlated
				Blood	Minimally invasive	Equal

RI = reverse iontophoresis.

## Data Availability

Not applicable.
